# Breast Cancer Cells Induce Cancer-Associated Fibroblasts to Secrete Hepatocyte Growth Factor to Enhance Breast Tumorigenesis

**DOI:** 10.1371/journal.pone.0015313

**Published:** 2011-01-13

**Authors:** Shiaw-Wei Tyan, Wen-Hung Kuo, Chun-Kai Huang, Chi-Chun Pan, Jin-Yuh Shew, King-Jen Chang, Eva Y.-H. P. Lee, Wen-Hwa Lee

**Affiliations:** 1 Genomics Research Center, Academia Sinica, Taipei, Taiwan Authority; 2 Department of Surgery, National Taiwan University Hospital, Taipei, Taiwan Authority; 3 Department of Development and Cell Biology, University of California Irvine, Irvine, California, United States of America; 4 Department of Biological Chemistry, University of California Irvine, Irvine, California, United States of America; The University of Hong Kong, Hong Kong, Special Administrative Region, People's Republic of China

## Abstract

It has been well documented that microenvironment consisting of stroma affects breast cancer progression. However, the mechanisms by which cancer cells and fibroblasts, the major cell type in stroma, interact with each other during tumor development remains to be elucidated. Here, we show that the human cancer-associated fibroblasts (CAFs) had higher activity in enhancing breast tumorigenecity compared to the normal tissue-associated fibroblasts (NAFs) isolated from the same patients. The expression level of hepatocyte growth factor (HGF) in these fibroblasts was positively correlated with their ability to enhance breast tumorigenesis in mice. Deprivation of HGF using a neutralizing antibody reduced CAF-mediated colony formation of human breast cancer cells, indicating that CAFs enhanced cancer cell colony formation mainly through HGF secretion. Co-culture with human breast cancer MDA-MB-468 cells in a transwell system enhanced NAFs to secret HGF as well as promote tumorigenecity. The newly gained ability of these “educated” NAFs became irreversible after continuing this process till fourth passage. These results suggested that breast cancer cells could alter the nature of its surrounding fibroblasts to secrete HGF to support its own progression through paracrine signaling.

## Introduction

During tumor progression, the stroma surrounding cancer cells has been found to undergo phenotypic and epigenetic changes [1], [2], [3], [4], [5]. The tumor stroma consists of a base membrane, extracellular matrix, blood vasculature, inflammatory cells and fibroblasts, which were all shown to contribute to cancer development [1], [6], [7]. Among these components, fibroblasts were found to have a predominant role in cancer progression [8], [9]. Fibroblasts in the breast tumor stroma were proposed to be “activated” to assist tumor development. Recent studies revealed that the primary cancer-associated fibroblasts (CAFs) derived from invasive breast carcinomas had greater potential to promote tumor growth and angiogenesis than the normal tissue-associated fibroblasts (NAFs) derived from non-cancer breast regions of the same patients or from reduction of mammoplasty tissues [10]. These results suggested that cancer cells may alter ability of neighboring fibroblasts to promote tumorigenesis. In addition, normal fibroblasts have also been shown to acquire oncogenic promoting activity by exposure to carcinogens, irradiation, wound healing and senescence [6]. These results indicated that under certain conditions the property of fibroblasts would be changed to assist tumor progression. However, the mechanism that makes these fibroblasts activated is not yet fully understood.

The communication between surrounding fibroblasts and cancer cells may go through cytokines. Previous reports revealed that gene expression profiles of myofibroblasts isolated from *in situ* and invasive breast carcinomas differed from those derived from normal breast tissues [11]. The differential expression included genes encoding secreted proteins and receptors, indicating that there are paracrine interactions between cancer cells and stromal myofibroblasts. Stromal cell-derived factor 1 (SDF-1/CXCL12) is one of the prominent chemokines secreted by stromal myofibroblasts. SDF-1 has been reported to be highly expressed in CAFs to promote tumorigenesis compared to NAFs [10]. In addition, hepatocyte growth factor (HGF) is an important fibroblast-secreted protein that mediates development and progression of cancers [12], [13]. HGF is mainly secreted from fibroblasts, whereas its receptor, c-Met receptor tyrosine kinase, is primarily expressed in epithelial cancer cells [14]. These results suggested that fibroblasts contribute to tumor development through secreting certain cytokine factors. However, whether the heterogeneous fibroblasts [15], [16] behave uniformly in response to heterogeneous cancer cells remains to be determined.

In this communication, we have compared five pairs of CAFs and NAFs derived from breast cancer patients. We found that NAFs have significantly lower tumor promoting activity compared to CAFs. In contrast to previous report, HGF, instead of SDF-1, is elevated in all CAFs. Deprivation of HGF by neutralizing with anti-HGF antibodies diminished the tumor promoting activity of CAFs. These results suggested that HGF may be a general contributing factor secreted from CAFs to promote tumorigenesis. Importantly, HGF expression and the tumor promoting activity of NAFs can be induced and fixed to similar levels as those of CAFs by long term co-culturing NAFs with breast cancer MDA-MB-468 cells in a transwell system. These finding provides evidence that breast cancer cells could induce alteration of fibroblasts via paracrine pathway to enhance fibroblast's ability to secrete HGF and promote tumorigenesis.

## Results

### Breast cancer-associated fibroblasts have higher ability to enhance breast tumorigenesis than normal tissue-associated fibroblasts

**Figure 1:**
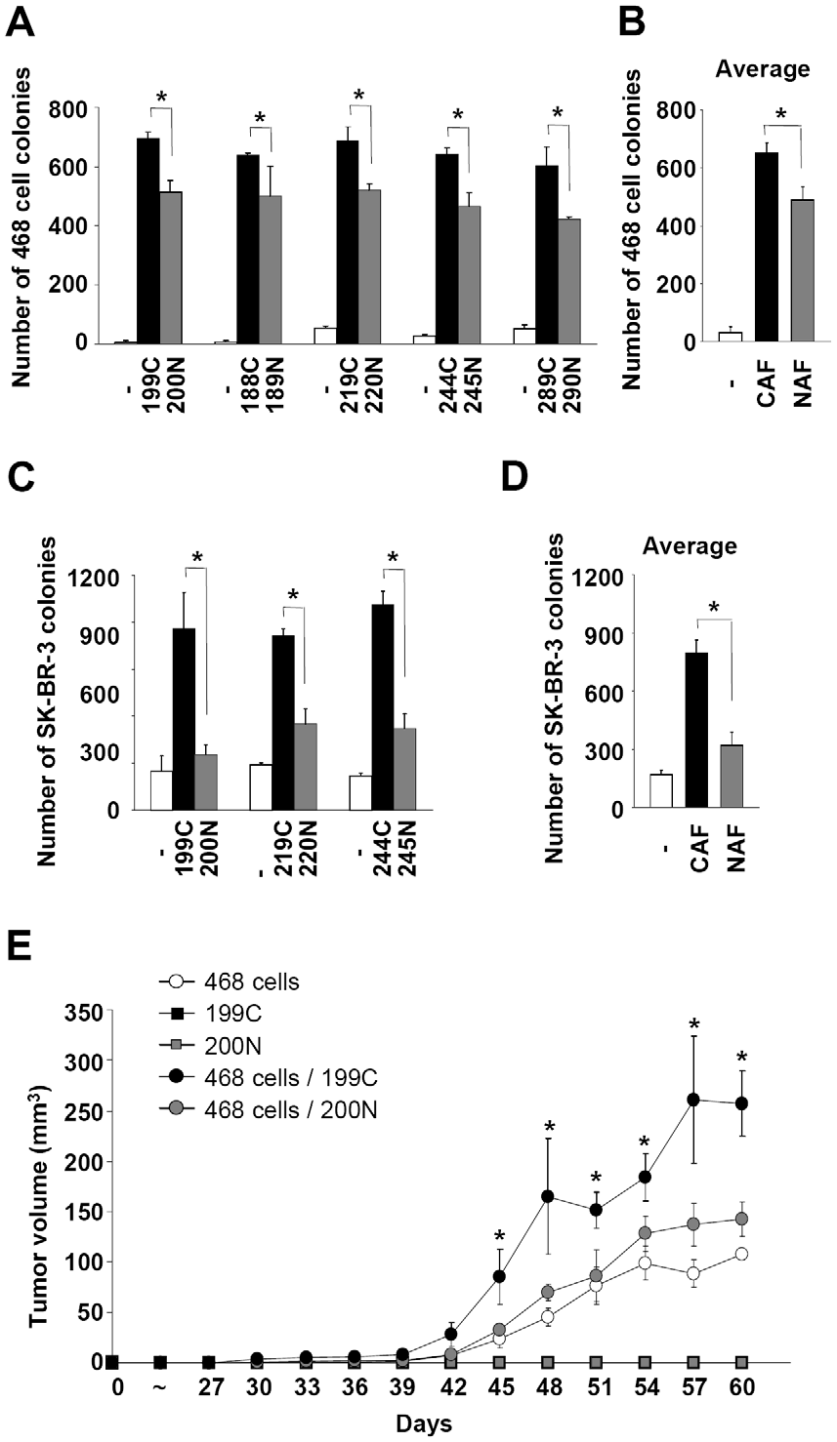
Breast cancer-associated fibroblasts enhanced breast tumorigenesis to a higher level than normal tissue-associated fibroblasts. (A) CAF/NAF pairs from the same patients were isolated and subjected to soft agar colony formation assay using MDA-MB-468 cells. For each pair of fibroblasts examined, CAFs significantly enhanced colony forming ability of MDA-MB-468 cells to a higher level than NAFs. Data are mean ± SD of triplicate samples. (B) The average of colony number of MDA-MB-468 cells mediated by CAFs and NAFs from all samples was shown. Data are mean ± SD. (C) For each pair of fibroblasts tested, CAFs enhanced soft agar colony forming ability of SK-BR-3 cells more effectively than NAFs. Data are mean ± SD of triplicate samples. (D) The average of SK-BR-3 cell colony numbers mediated by CAFs and NAFs from all samples was shown. Data are mean ± SD. (E) CAF #199C significantly enhanced tumor growth in the NOD/SCID fat pads than its normal counterpart NAF #200N and the control (no fibroblasts). Tumor volume was determined every three days after injection. Data are mean ± SEM of tumors from 6 mice. Statistical significance between CAF #199C and NAF #200N was evaluated by Student's t-test. * *P*<0.05.

To compare the differential effects of CAFs and NAFs on breast tumorigenesis, we isolated fibroblasts of human breast cancer tissue and adjacent normal breast tissue from the same patients. These primary fibroblasts were grown to 100% confluent in culture and then evaluated for their abilities to promote cancer cells to form colony in soft agar. Using this soft agar colony formation system, we compared the effects of five pairs of CAFs and NAFs on the MDA-MB-468 cell colony formation in nutrition restricted medium, in which MDA-MB-468 cells could not form colonies in the absence of fibroblasts. Although both CAFs and NAFs were able to support MDA-MB-468 cells to form colonies, significantly more colonies (about 30–50% more) were formed when cells were co-cultured with CAFs (with the average about 650 colonies) compared to NAFs co-culture (about 490 colonies) (Figure 1A and 1B). Similar results were observed using another breast cancer cell line, SK-BR-3 (Figure 1C and 1D). Taken together, these results indicated that CAFs, compared to NAFs, significantly enhanced colony formation of these breast cancer cells.

Next, we examined whether CAFs and NAFs differentially support tumor growth *in vivo* using immunocompromised NOD/SCID mice. Fibroblasts from 100% confluent culture were mixed with MDA-MB-468 cells at 5∶1 ratio and injected into the fat pads of the NOD/SCID mice. CAF #199C significantly enhanced the tumor growth by 2-3-fold compared to NAF #200N and the control (no fibroblasts) (Figure 1E). CAFs or NAFs alone did not lead to tumor formation. The tumors were composed of lots of MDA-MB-468 cells and very few human fibroblasts three weeks after injection. Thus, CAFs possessed higher ability to enhance the breast tumorigenesis *in vivo* at the initial stage than NAFs.

### Identification of secreted factors from CAFs and NAFs that affect breast tumorigenesis

**Figure 2:**
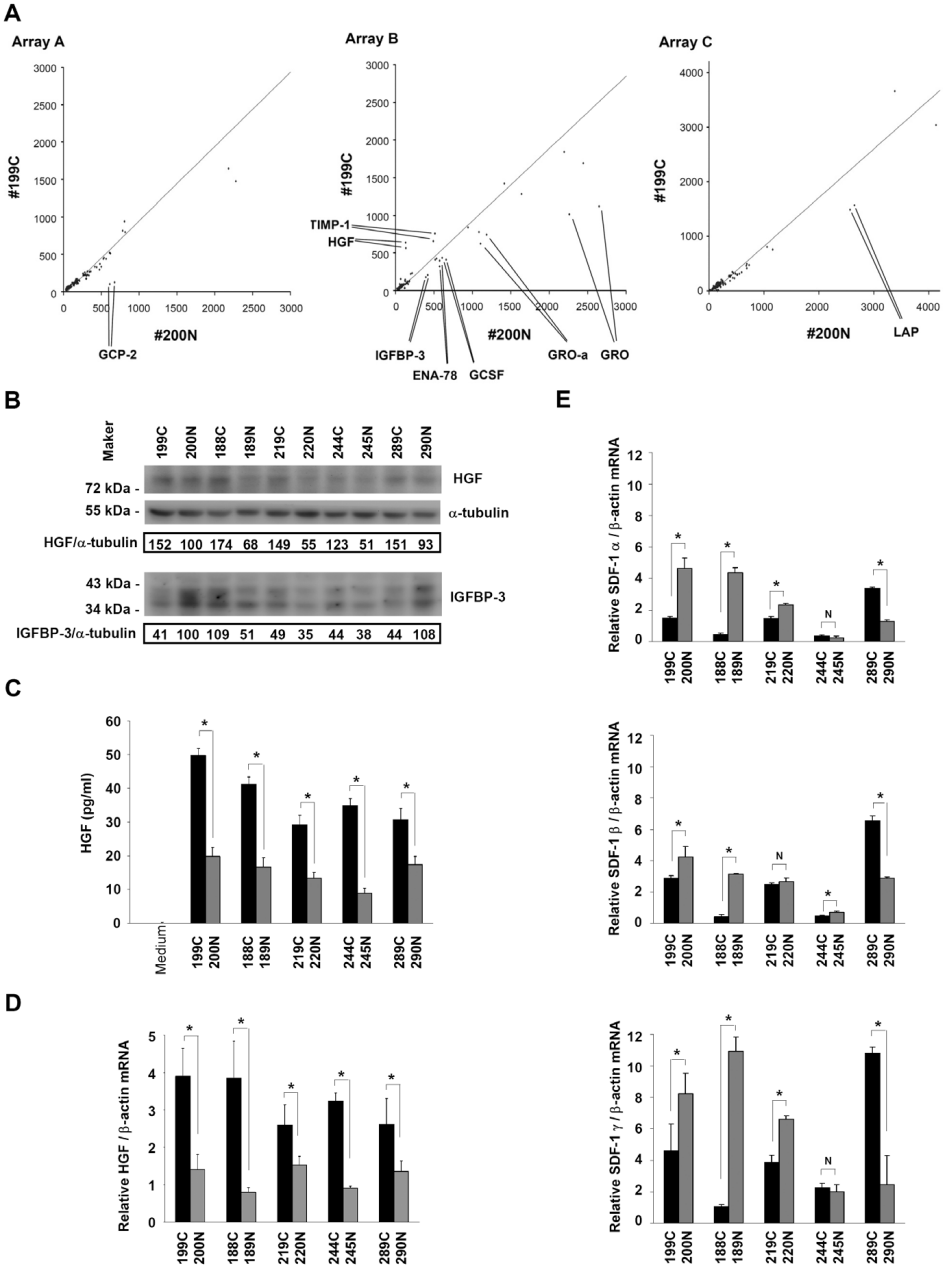
Breast cancer-associated fibroblasts expressed higher HGF levels than normal tissue-associated fibroblasts. (A) Regression of the cytokine/growth factor signal intensities in the conditional media from CAF #199C and NAF #200N was performed to evaluate the difference of the cytokines and growth factors secreted by CAFs and NAFs. The results revealed that HGF (4.57 fold) and TIMP-1 (1.41 fold) levels were significantly higher in the conditional medium from CAFs than those in the conditional medium from NAFs (array B). In contrast, lower levels of GCP-2 (0.17 fold) (array A), IGFBP-3 (0.48 fold), GRO family (0.44 fold), GRO-α (0.6 fold), ENA-78 (0.63 fold), GCSF (0.54 fold) (array B) and LAP (0.59 fold) (array C) were detected in the conditional media from CAFs compared to those in the conditional medium from NAFs. (B) Western blotting analysis revealed that the HGF protein levels in CAFs were higher than those in NAFs in all fibroblast pairs (upper panel). Whereas, there was no correlation observed between CAFs and IGFBP-3 protein expression (lower panel). (C) Enzyme-linked immunosorbent assay showed that the HGF amounts in the cultured media of CAFs were higher than those of NAFs in all fibroblast pairs. (D) Quantitative real-time RT-PCR analysis showed that the HGF mRNA levels in CAFs were higher than those in NAFs in all fibroblast pairs. (E) Real-time RT-PCR analysis revealed that the mRNA levels of SDF-1 α, SDF-1 β and SDF-1 γ varied in different samples. There was no association between the SDF-1 mRNA levels and CAFs. Data are mean ± SD of triplicate samples. * *P*<0.05. NS, no significant difference.

To identify the factors that contribute to or inhibit tumorigenesis, we compared the profiles of secreted proteins from CAF #199C and NAF #200N using the cytokine/growth factor antibody arrays. The results showed that hepatocyte growth factor (HGF) and tissue inhibitor of metalloproteinases-1 (TIMP-1) (examined by the array B) were secreted at higher levels from CAFs compared to NAFs (Figure 2A and Figure S1). In contrast, granulocyte chemotactic protein-2 (GCP-2; also named CXCL6, CXC ligand 6) (examined by the array A), insulin-like growth factor binding protein-3 (IGFBP-3), growth-related oncogene (GRO; the antibody could recognizes GRO-α, GRO-β and GRO-γ), growth-related oncogene-alpha (GRO-α; also named CXCL1, CXC ligand 1), epithelial cell-derived neutrophil-activating peptide-78 (ENA-78; also named CXCL5, CXC ligand 5), granulocyte colony stimulatory factor (GCSF) (the array B) and latency-associated peptide (LAP) (the array C) were secreted at lower levels from CAFs compared to NAFs. The levels of monocyte chemotactic protein-1 (MCP-1; also named CCL2), insulin-like growth factor binding protein-6 (IGFBP-6) and insulin-like growth factor-II (IGF-II) were identical from both fibroblasts and were used as the internal control.

To confirm the results of the cytokine/growth factor antibody arrays, Western blotting analysis for several growth factors was carried out. The HGF protein levels in CAFs were higher than those in NAFs (Figure 2B). The HGF protein levels secreted from CAFs were also higher than those from NAFs, assessed by enzyme-linked immunosorbent assay (Figure 2C). These finding were consistent with the results of the cancer cell colony formation assays and the tumorigenesis assay in the NOD/SCID mice. The results of Western blotting analysis also indicated that the levels of IGFBP-3 in CAFs and NAFs were not correlated to fibroblast-mediated tumor growth.

The messenger RNA (mRNA) levels of HGF in CAFs and NAFs were also compared by quantitative real-time RT-PCR analysis. The results showed that the HGF mRNA levels in CAFs were higher than NAFs (Figure 2D), indicating that differences in the HGF protein levels were likely due to alterations in the mRNA. The mRNA level of SDF-1, which was proposed to play an essential role in stromal fibroblast-mediated breast tumorigenesis [10], was also examined. However, there was no strict correlation between the mRNA levels of SDF-1 α, β and γ isoforms and the abilities of these fibroblasts to promote breast tumorigenesis (Figure 2E).

### HGF neutralizing antibodies abolished soft agar colony-promoting effect of CAFs

**Figure 3:**
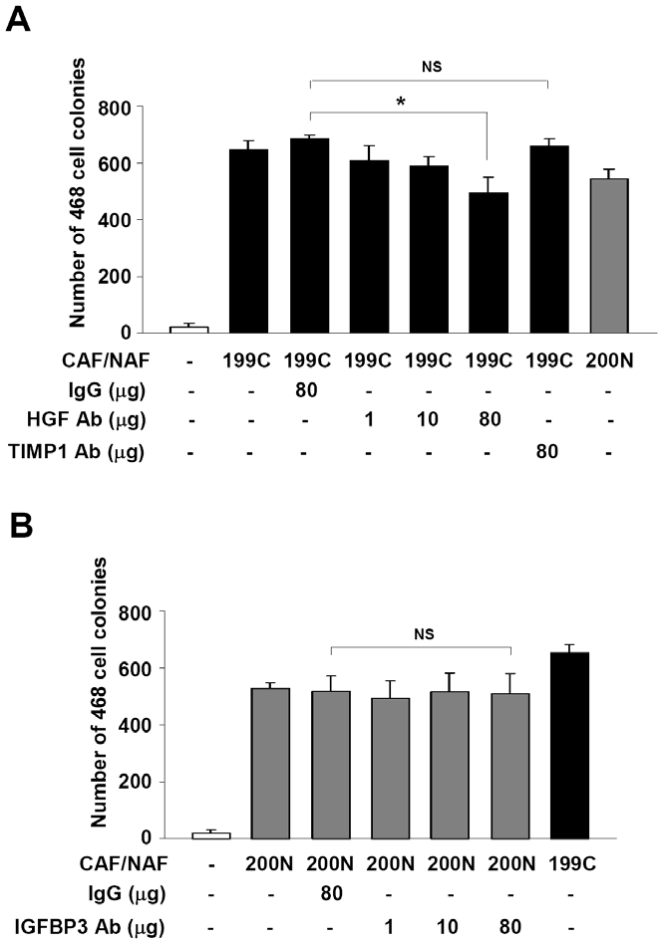
Sequestration of the HGF activity reduced cancer-associated fibroblast-mediated soft agar colony formation of MDA-MB-468 cells. (A) Neutralization of HGF activity by addition of 80 µg/ml anti-HGF antibody significantly reduced CAF #199C-mediated soft agar colony formation of the MDA-MB-468 cells. However, the anti-TIMP-1 antibody did not show any effect. (B) NAF #200N-mediated soft agar colony formation of MDA-MB-468 cells was not affected by addition of 80 µg/ml anti-IGFBP-3 antibody. Data are mean ± SD of three independent experiments. * *P*<0.05. NS, no significant difference.

To test whether HGF directly contributes to the effect on enhancing breast cancer cells in soft agar colony formation, anti-HGF antibodies were added to the soft agar medium to neutralize the HGF activity. The colony number of the MDA-MB-468 cells in the CAF #199C co-culture was significantly reduced to the level similar to NAF #200N by the addition of 80 µg/ml of the anti-HGF antibody (Figure 3A). However, the MDA-MB-468 cell colony formation was not affected by the addition of antibodies against TIMP-1. Also, the number of the MDA-MB-468 cell colonies supported by NAF #200N was unaffected when an anti-IGFBP-3 antibody up to the concentration of 80 µg/ml was added (Figure 3B). Overall, our results suggested that HGF is the major factor contributing to the differential effects of CAFs on cancer cell colony forming ability.

### Pre-coculture with breast cancer cells enhanced the ability of NAFs to mediate cancer cell colony formation and breast tumor growth

**Figure 4:**
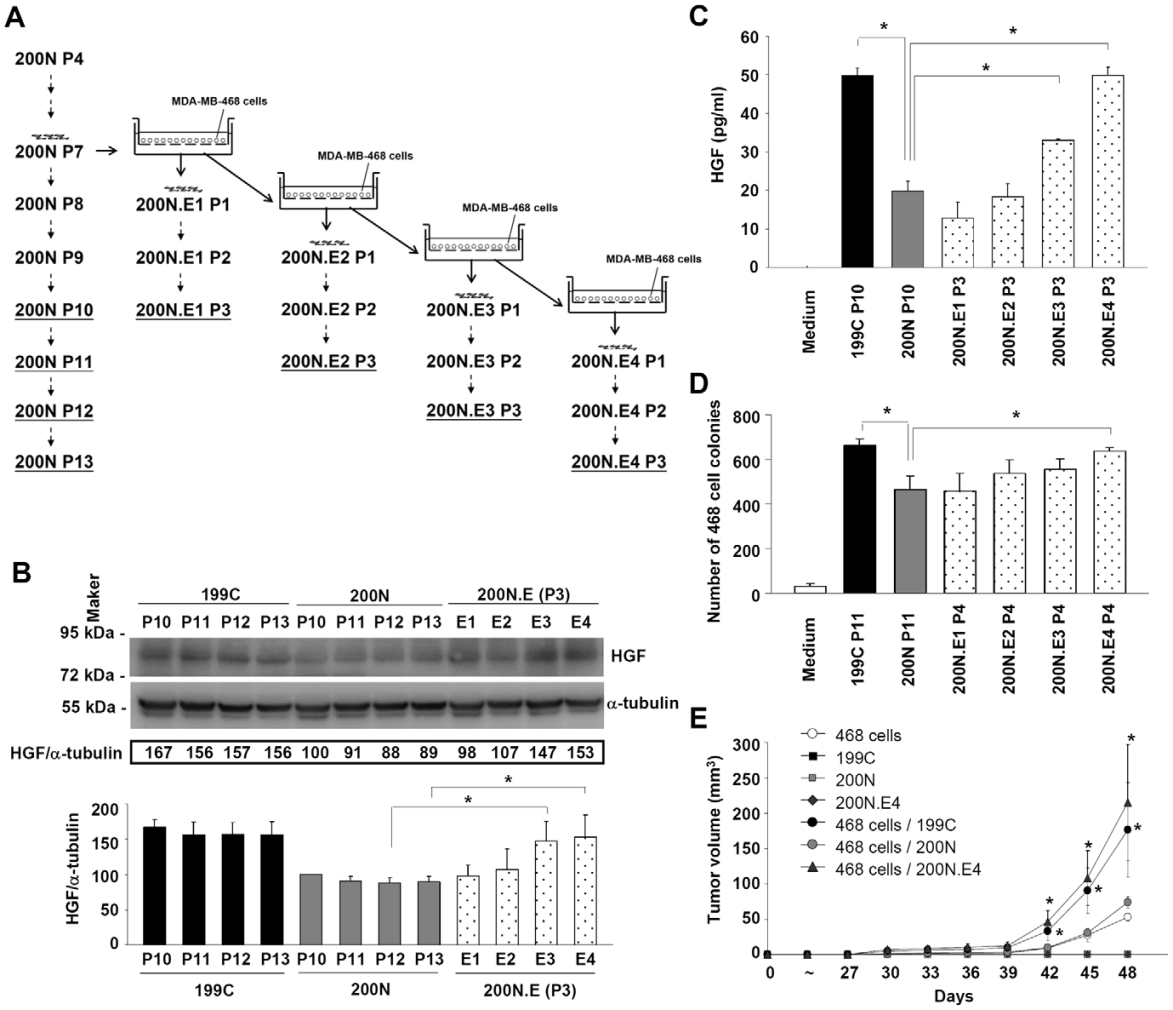
Pre-coculture with breast cancer cells enhanced the ability of normal tissue-associated fibroblasts to mediate breast tumorigenesis. (A) The protocol of the co-culture system using MDA-MB-468 cells and NAFs was shown. NAF #200N co-cultured with MDA-MB-468 cells for four passages was indicated as 200N.E1-E4, respectively. Each fibroblast of 200N.E1-E4 was propagated in the absence of MDA-MB-468 cell co-culture and passaged from P.1 to P3. (B) The HGF protein expression in NAF #200N.E1-E4 was shown. The HGF protein levels in NAF #200N.E1 and E2 were reduced to the level in NAF #200N after three passages (P.3). However, the HGF protein levels in NAF #200N.E3 P.3 and E4 P.3 were significantly higher than those in NAF #200N. (C) Enzyme-linked immunosorbent assay showed that the HGF protein amounts in the cultured media of NAF #200N.E3 P.3 and E4 P.3, rather than E1 P.3 and E2 P.3, were higher than those of NAF #200N. (D) NAF #200N.E4 P.4 significantly enhanced soft agar colony formation of MDA-MB-468 cells to a higher level than NAF #200N. For (B), (C) and (D), data are mean ± SD of three independent experiments. (E) NAF #200N.E4 P.4 significantly increased the tumor growth in the NOD-SCID fat pads compared to NAF #200N. Tumor volume was determined every three days after injection. Data are mean ± SEM of tumors from 4 mice. Difference between NAF #200N.E4/CAF #199C and NAF #200N was evaluated by Student's t-test. * *P*<0.05.

To test whether cancer cells instruct surrounding fibroblasts to secrete factors such as HGF to promote tumor growth, we co-cultured NAF #200N with MDA-MB-468 cells using a transwell insert containing a 0.4-µm polyester membrane. In this co-culture system, NAFs were grown on the bottom of the culture dish and MDA-MB-468 cells were seeded on the membrane of the transwell insert. After incubation for 3.5 days, NAF #200N was isolated (indicated as NAF #200N.E1) and propagated in the absence of MDA-MB-468 cells for further passages (indicated as NAF #200N.E1 P.1, P.2 and P.3, respectively); or continued to co-culture with MDA-MB-468 cells to generate NAF #200N.E2 (Figure 4A). Following this scheme, we also generated NAF #200N.E3 and NAF #200N.E4. The HGF protein levels in NAF #200N.E1-E4 co-cultured with the MDA-MB-468 cells were increased, compared to those in NAF #200N when analyzed by Western blotting (Figure S2). The HGF protein levels in NAF #200N.E1 and E2 were reduced to the level of NAF #200N up to the third passage in the absence of MDA-MB-468 cell co-culture (Figure 4B). However, the HGF protein levels in NAF #200N.E3 P.3 and E4 P.3 were still higher than those in NAF #200N. The HGF amounts secreted from NAF #200N.E3 P.3 and E4 P.3, but not E1 P.3 and E2 P.3, were also higher than NAF #200N (Figure 4C). These results indicated that co-culture with MDA-MB-468 cells was sufficient to induce HGF protein secretion from NAFs. However, stabilization of HGF induction required prolonged co-culture since subcultures of NAF #200N.E1 and E2 did not have a sustained elevated HGF protein level.

To examine whether NAFs, pre-cocultured with MDA-MB-468 cells, increase their capacities to support tumor growth, we performed colony formation assays to evaluate the colony formation promotion abilities of NAF #200N.E1-E4 P.4 compared to NAF #200N. The results showed that NAF #200N.E4 P.4 exhibited higher promoting ability than NAF #200N (Figure 4D). However, NAF #200N.E1-E3 P.4 did not significantly increase the MDA-MB-468 cell colony formation than NAF #200N. These results indicated that pre-coculture with MDA-MB-468 cells for 4 passages, rather than 3 passages, stimulated tumorigenic promoting ability of NAFs. HGF protein levels in NAF #200N.E3 P.3 and E4 P.3 were both significantly higher than NAF #200N. Thus, it is likely that the expression of HGF in NAF #200N E3 P.3 cannot sustain to promote the MDA-MB-468 cell colony formation during the assay. Consistently, in breast tumorigenesis analysis using the NOD/SCID mice, NAF #200N.E4 P.4 significantly promoted the MDA-MB-468 tumor growth compared to NAF #200N (Figure 4E). Taken together, these results suggested that coculture with the breast cancer cells could change the nature of NAFs to facilitate breast tumorigenesis.

## Discussion

In this communication, we evaluated the differential contribution of CAFs and their counterpart NAFs derived from the same breast cancer patients to breast tumorigenesis. We found that CAFs had higher ability to promote breast cancer MDA-MB-468 cells to form colonies in soft agar and to facilitate tumor growth in NOD/SCID mice than NAFs. By comparing the profiles of proteins secreted from CAFs and NAFs using the cytokine/growth factor antibody arrays, significantly higher levels of HGF and TIMP-1 secreted from CAFs were revealed. The amount of HGF, but not TIMP-1, from these fibroblasts was positively correlated with their ability to enhance breast tumorigenesis. Conversely, deprivation of HGF using a neutralizing antibody reduced CAF-mediated colony formation of breast cancer cells, indicating that CAFs enhanced cancer cell colony formation mainly through HGF. Co-culturing NAFs with breast cancer cells in a transwell system for several passages was able to enhance the ability of NAFs to promote tumorigenicity as well as HGF expression to the compatible level as that of CAFs. These results indicated that breast cancer cells reprogram its surrounding fibroblasts to secrete HGF, in part, to support its own progression via paracrine signaling.

HGF secreted by fibroblasts has been shown to mediate proliferation and invasion of cancer cells [12], [13]. It has been reported that HGF is primarily expressed and secreted from fibroblasts [8], [17]. Consistently, we found that HGF expression level was extremely low in breast cancer MDA-MB-468 cells compared to CAFs and NAFs (Figure S3). Ectopic expression of HGF in breast fibroblasts was used to promote tumor initiation and growth of human breast epithelial organoids in the humanized fat pads of NOD/SCID mice [18], [19]. Consistently, our data revealed that HGF protein and mRNA levels in fibroblasts derived from the breast cancer patients were positively correlated with their abilities to enhance breast tumorigenesis. Neutralization of HGF activity reduced CAF-mediated colony formation of breast cancer cells, suggesting that HGF secreted by CAFs may be the major contributing factor for this differential tumorigenic promoting ability between CAFs and NAFs. Although TIMP-1 was the other factor significantly elevated in CAF cells, deprivation of TIMP-1 activity did not affect cancer cell colony formation promoted by CAFs (Figure 3A). It is likely that the soft agar colony assay system may fail to address the contribution of TIMP-1 in tumorigenesis because the main function of TIMP-1 is to inhibit activities of matrix metalloproteinases (MMPs) [20]. Intriguingly, SDF-1 released by stroma fibroblasts has been reported as a major factor contributing to breast tumorigenesis [10]. However, our data showed that the SDF-1 mRNA levels in those fibroblasts were not strictly correlated with their abilities to mediate breast tumorigenesis (Figure 2D), indicating that HGF, rather than SDF-1, is a common contributing factor of CAFs in promoting tumorigenesis.

Based on the cytokine array assay, the levels of GCP-2, GRO-α, ENA-78, IGFBP-3 and LAP secreted from CAFs were lower than NAFs (Figure 2A). IGFBP-3 has been shown to be an invasion suppressor [21]. Whether it plays a role in cancer cell proliferation and tumor growth remains unclear. Our data suggested that neutralizing IGFBP-3 activity did not affect cancer cell colony formation promoted by NAFs (Figure 3B), indicating that IGFBP-3 may not be the contributing factor in this soft agar assay system. Similarly, blockage of GCP-2 activity did not affect NAF-mediated colony formation of cancer cells (Figure S4), although GCP-2, GRO-α and ENA-78 belong to the CXC chemokine family, possibly involved in metastasis and tumor growth [22]. LAP is known to be associated with transforming growth factor-beta (TGF-β) to form a latent TGF-β complex [23], which is secreted and activated by binding to latent TGF-beta binding protein (LTBP). However, whether LAP plays a role in tumorigenesis remains to be tested.

The differential effects derived from CAFs and NAFs suggest that cancer cells have the ability to instruct their surrounding fibroblasts to reprogram their expression profiles to support cancer cell growth. To directly address this possibility, we used a transwell system to co-culture NAFs with breast cancer cells to test how NAFs respond to cancer cells. Using HGF as a common marker, it appeared that pre-coculture with the breast cancer MDA-MB-468 cells increased and maintained higher HGF protein levels in NAF #200N.E3 and E4 (Figure 4B and 4C). Importantly, high level of HGF in NAF #200N E4 exhibited significantly higher ability to enhance cancer cell colony formation than NAFs #200N. These results revealed a process that cytokine factors secreted from breast cancer cells induced NAFs to secrete HGF in spite of those factors were removed.

How breast cancer cells instruct their surrounding fibroblasts to promote tumor progression is not fully understood. Platelet-derived growth factor (PDGF), fibroblast growth factor 2 (FGF2) and TGF-β released by cancer cells may be candidates to mediate fibroblasts activation, but the previous data were not sufficient to support this notion [9]. For example, TGF-β could play a role in suppressing the ability of fibroblasts to mediate cancer initiation [8], [9]. Conditional inactivation of TGF-β type II receptor in fibroblasts in mice was shown to induce epithelial cells in prostate and fore stomach to malignancy, which may be regulated by elevation of HGF secretion from fibroblasts [24]. These results indicated that TGF-β signaling may suppress secretion of HGF from fibroblasts to limit proliferation of adjacent epithelial cells in normal condition. However, whether TGF-β signaling is blocked in activated fibroblasts or not and how HGF or other factors secreted by activated fibroblasts make epithelial cells to become malignant remain to be elucidated. Nevertheless, the factors secreted from co-cultured breast cancer cells via paracrine signaling will induce the surrounding fibroblasts to change the expression profiles. The fact that tumor promoting ability of instructed fibroblasts remains even the factors from cancer cells were removed, suggested a genome reprogramming in these fibroblasts. The established co-culture system will allow us to further dissect this reprogramming process.

## Materials and Methods

### Ethics statement

All human specimens were encoded to protect patient confidentiality and processed under protocols approved by the Institutional Reviews Board of Human Subjects Research Ethics Committee of Academia Sinica (AS-IRB02-98042) and National Taiwan University hospital (#200902001R), Taipei, Taiwan. Breast cancer tissues and its relative normal counterparts were obtained from patients who underwent surgery at National Taiwan University Hospital. Signed consent for the studies was obtained from all the patients.

Animal care and experiments were approved by the Institutional Animal Care and Utilization Committee of Academia Sinica (IACUC #080085).

### Clinical specimens and cell cultures

All human breast cancer tissues and its relative normal counterparts were minced to 2–3 mm^3^ cubes and attached onto the culture dishes for fibroblast culture. Primary fibroblast isolated from clinical specimen were maintained in Dulbecco's Modified Eagle's Medium/F-12 Nutrient Mixture (DMEM/F-12) (Invitrogen) supplemented with 10% fetal bovine serum (Industrial Biological), 0.1 mM non-essential amino acids and 1 mM sodium pyruvate (Invitrogen, above). Breast cancer cell lines, MDA-MB-468 and SK-BR-3, were cultured in DMEM/F-12, supplemented with 10% fetal bovine serum. Cells were incubated at 37°C in a humidified incubator with 5% CO_2_.

### Soft agar colony formation assay

2×10^5^ primary fibroblasts were seeded in a 35-mm culture dish and cultured for 3–4 days to reach 100% confluent. Cells were further incubated one more day before assay. After washed with phosphate buffered saline twice, 1 ml of 0.5% agar in DMEM/F-12 containing 2% fetal bovine serum was added on top of the fibroblasts to form a base layer. For neutralization of cytokines and growth factors, mouse anti-human HGF, anti-human TIMP-1 and anti-human IGFBP-3 antibodies (R&D Systems) were added into the medium and mixed with the agar. After the agar was solidified, 5000 MDA-MB-468 or SK-BR-3 cells were evenly suspended in 1 ml of 0.35% agar in DMEM/F-12 containing 2% fetal bovine serum and then added into the dish to form a cancer cell layer. Dishes were incubated in a humidified, 37°C, 5% CO_2_ incubator until cell colonies appear obviously (11 days for MDA-MB-468 cells and 21 days for SK-BR-3 cells). Colonies were fixed with 0.05% Crystal violet solution and counted (diameter larger than 40 µm) under light microscopy.

### Breast tumorigenesis assay in NOD/SCID mice

Five to six-week-old female immunocompromised NOD/LtSz-*scid* mice (The Jackson Laboratory) were used for *in vivo* tumorigenesis assay. 5×10^4^ MDA-MB-468 cells were mixed with 2.5×10^5^ CAFs/NAFs with passage number 4–6, and then mixed with Matrigel matrix (BD Biosciences) before injected into the fourth mammary fat pads of the mice. The tumors of the MDA-MB-468 cells were examined and determined for the volume up to two months. Mice were housed in a room maintained on a 12 h light/dark cycle (light on at 6 a.m.) with food and water provided *ad libitum*.

### Cytokine/growth factor antibody array analysis

The conditional media from CAF #199C and NAF #200N, which contain no fetal bovine serum, were collected after incubation for 24 hours. The conditional media were further concentrated by the centrifugal filter devices (Amicon Ultra-4, 3 k, Millipore) to 100-fold concentrated volume and then subjected to the human cytokine antibody arrays (G series 2000, RayBiotech). These arrays included array G series 6 (array A), array G series 7 (array B) and array G series 8 (array C). 5 µl (0.05 µg of protein content) of conditional medium was added to each cytokine antibody array to perform the assay following manufacturer's procedures.

### Western blotting analysis

Fibroblasts were grown to 100% confluent and replaced with fresh medium one day before protein extraction with radio-immunoprecipitation assay (RIPA) buffer containing 50 mM Tris-HCl (pH 7.4), 150 mM NaCl and 1% IGEPAL CA-630, 2 mM EDTA and protease inhibitors with thorough homogenization. Lysates were clarified by centrifugation and resolved by SDS-8% PAGE with 30 µg of protein content. The proteins resolved by SDS-PAGE were transferred to the PVDF membrane and immunoblotted with antibodies including mouse monoclonal anti-human HGF, anti-human IGFBP-3 (R&D Systems, above), and anti-alpha tubulin (abcam) antibodies followed by a horseradish peroxidase (HRP)-conjugated horse-anti-mouse IgG antibody (Cell Signaling). Membrane was developed by reacting with chemiluminescenct HRP substrate and exposed to BioSpectrumAC imaging system (Ultra-Violet Products).

### Enzyme-linked immunosorbent assay

Fibroblasts were grown to 100% confluent and replaced with fresh medium one day before assay. The cultured medium was clarified by centrifugation at 300× g for 5 min. 100 µl of supernatant was subjected to enzyme-linked immunosorbent assay for HGF (RayBiotech) following the manufacturer's instruction.

### Quantitative real-time RT-PCR

Total RNA from fibroblasts was isolated using TRI reagent (Ambion). Purified RNA (1 µg) was subjected to cDNA synthesis by Superscript II reverse transcriptase (Invitrogen) in 20 µl of reaction volume. Real-time RT-PCR analysis was performed using the ABI PRISM 7000 sequence detection system with SYBER Green method (Applied Biosystems) according to the manufacturer's instruction. The primers used in this assay were: HGF: 5′-AGT TGG CTA CTG CTC CCA AA- 3′ and 5′-TTC CAT GTT CTT GTC CCA CA-3′; SDF-1 α: 5′-TGA GAG CTC GCT TTG AGT GA- 3′ and 5′-CAC CAG GAC CTT CTG TGG AT-3′; SDF-1 β: 5′-CTA GTC AAG TGC GTC CAC GA- 3′ and 5′-GGA CAC ACC ACA GCA CAA AC-3′; SDF-1 γ: 5′-GTG CCC TTC AGA TTG TAG CC- 3′ and 5′-GGG CAG CCT TTC TCT TCT TC-3′; the internal control, beta-actin: 5′-ATC TGG CAC CAC ACC TTC TAC A- 3′ and 5′-TCA CCG GAG TCC ATC ACG AT- 3′. The amplification mixture contained 1 µl of 5× diluted reverse transcription product, 200 nM of each primer, 250 nM probe, and 12.5 µl of 2× SYBER Green PCR master mix (Applied Biosystems) in a total of 20-µl reaction volume. The thermal conditions were: 2 min at 50°C and 10 min at 95°C followed by 40 cycles at 95°C for 15 sec and 55°C for 1 min. The relative quantity of mRNA was estimated by using a standard curve created by serial dilution of the reverse transcription products from NAFs. Semi-quantitative analysis of the HGF gene expression was normalized to that of the beta actin gene expression.

### Co-culture of fibroblasts with cancer cells

In the co-culture system, 8×10^4^ NAF #200N P.7 were grown in the bottom of a 6-well plate in 2.5 ml of DMEM/F-12 with 10% fetal bovine serum and 1×10^5^ MDA-MB-468 cells were seeded on the 0.4-µm polyester membrane of a transwell insert (Corning) in 1.5 ml of the same medium. Dishes were incubated in a humidified, 37°C, 5% CO_2_ incubator. NAF #200N.E1 was derived after incubation for 78 hours. Some of NAF #200N.E1 fibroblasts were subcultured and grown for generation of P.1, P.2 and P.3 without MDA-MB-468 cell co-culture; others were subcultured and continued to co-culture with MDA-MB-468 cells to generate NAF #200N.E2 after 78 hours. Following the same procedure, NAF #200N.E3 and NAF #200N.E4 were obtained, and all the fibroblasts with passage number P.1, P.2 and P.3 were also generated.

## Supporting Information

Figure S1 **Cytokine/growth factor antibody array analysis of the conditional media from CAFs and NAFs.** Image of the cytokine/growth factor antibody array revealed that HGF and TIMP-1 levels were significantly higher in the conditional medium from CAF #199C than those in the conditional medium from NAF #200N (array B). In contrast, lower levels of GCP-2 (array A), IGFBP-3, GRO family, GRO-α, ENA-78, GCSF (array B) and LAP (array C) were detected in the conditional media from CAF #199C compared to those in the conditional medium from NAF #200N. The identical levels of MCP-1, IGFBP-6 and IGF-II were used as the internal control for each array.
(TIF)

Figure S2 **Co-culture with breast cancer MDA-MB-468 cells enhanced the HGF protein expression in NAFs.** Western blotting analysis revealed that the HGF protein levels in MDA-MB-468 cell-cocultured NAF #200N.E1-E4 were higher than NAF #200N. Data are mean ± SD of three independent experiments.
(TIF)

Figure S3 **Breast cancer MDA-MB-468 cells expressed low level of HGF.** Real-time RT-PCR analysis showed that HGF expression in MDA-MB-468 cells was extremely low compared to CAF #199C and NAF #200N. Data are mean ± SD of triplicate samples. * *P*<0.05. *** *P*<0.001.
(TIF)

Figure S4 **Sequestration of the GCP-2 activity did not affect NAF-mediated soft agar colony formation of the breast cancer MDA-MB-468 cells.** NAF #200N-mediated soft agar colony formation of MDA-MB-468 cells was not affected by addition of 80 µg/ml anti-GCP-2 antibody. Data are mean ± SD of triplicate samples. * *P*<0.05. NS, no significant difference.
(TIF)
