# Supplementary figures and images for: Breast Cancer Cells Induce Cancer-Associated Fibroblasts to Secrete Hepatocyte Growth Factor to Enhance Breast Tumorigenesis
Source: PLoS One. 2011 Jan 13;6(1):e15313. doi: 10.1371/journal.pone.0015313 (PMC3020942; doi:10.1371/journal.pone.0015313)

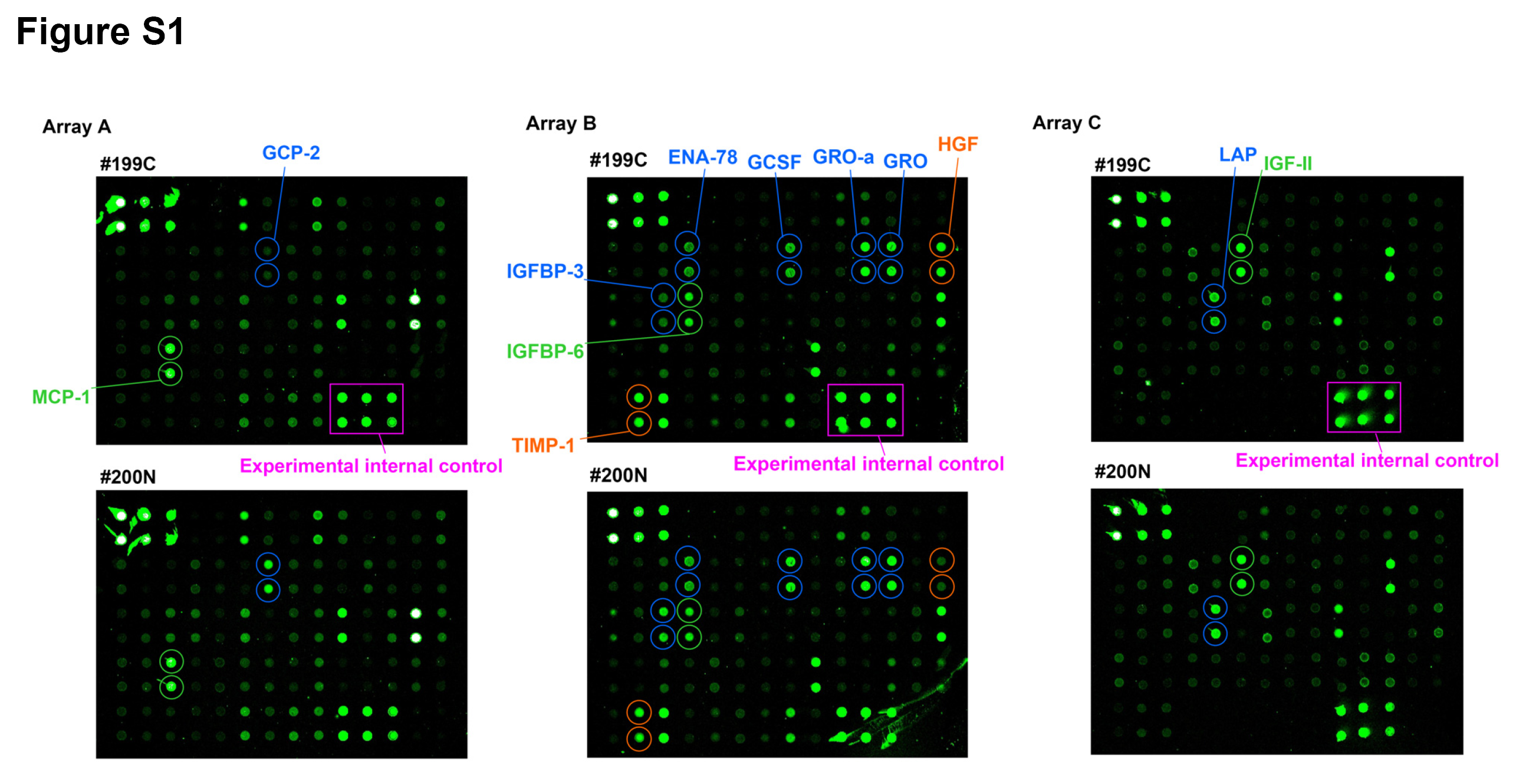

Supplement: Figure S1 — Cytokine/growth factor antibody array analysis of the conditional media from CAFs and NAFs. Image of the cytokine/growth factor antibody array revealed that HGF and TIMP-1 levels were significantly higher in the conditional medium from CAF #199C than those in the conditional medium from NAF #200N (array B). In contrast, lower levels of GCP-2 (array A), IGFBP-3, GRO family, GRO-α, ENA-78, GCSF (array B) and LAP (array C) were detected in the conditional media from CAF #199C compared to those in the conditional medium from NAF #200N. The identical levels of MCP-1, IGFBP-6 and IGF-II were used as the internal control for each array. (TIF) [file pone.0015313.s001.tif]

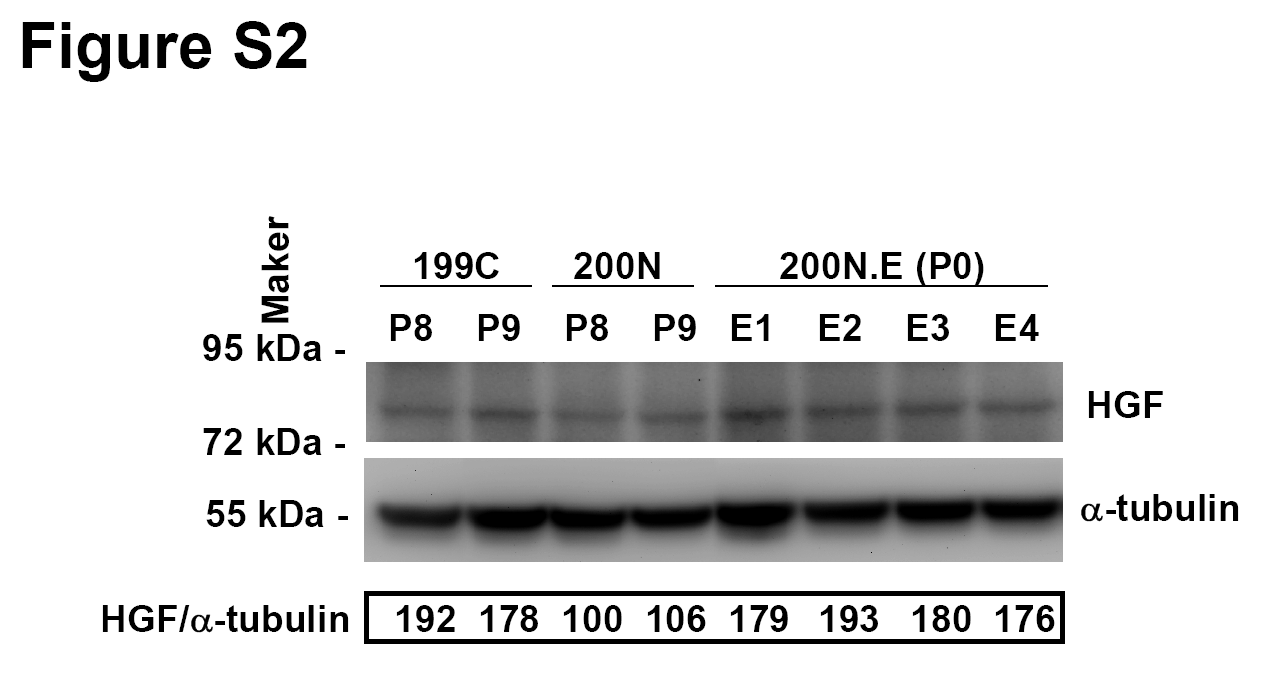

Supplement: Figure S2 — Co-culture with breast cancer MDA-MB-468 cells enhanced the HGF protein expression in NAFs. Western blotting analysis revealed that the HGF protein levels in MDA-MB-468 cell-cocultured NAF #200N.E1-E4 were higher than NAF #200N. Data are mean ± SD of three independent experiments. (TIF) [file pone.0015313.s002.tif]

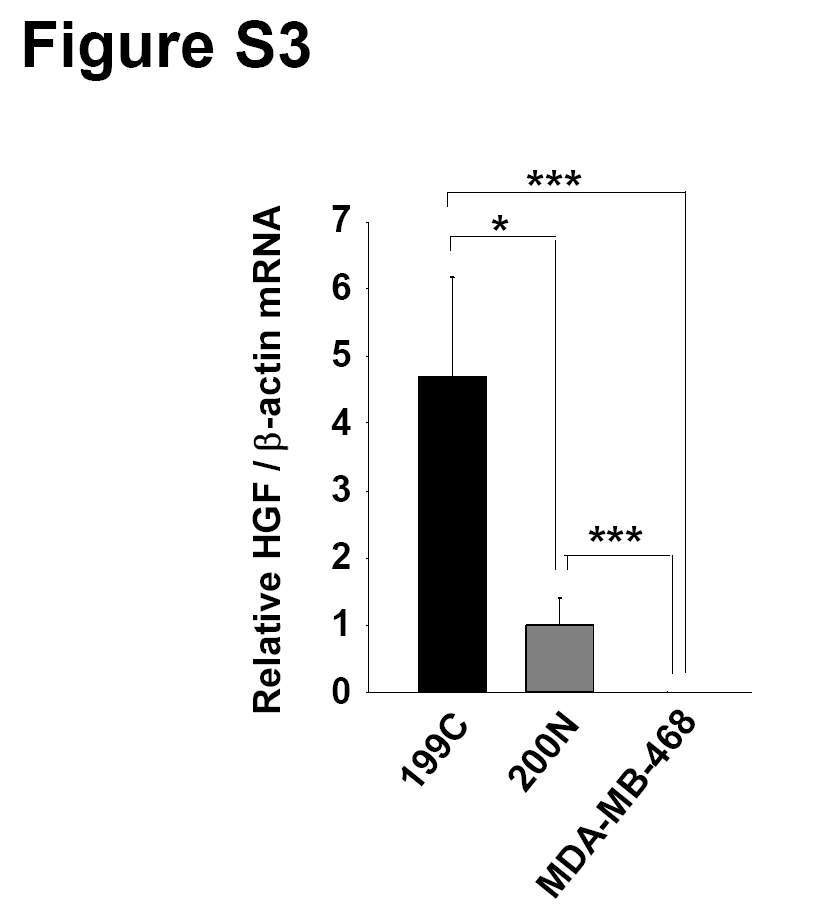

Supplement: Figure S3 — Breast cancer MDA-MB-468 cells expressed low level of HGF. Real-time RT-PCR analysis showed that HGF expression in MDA-MB-468 cells was extremely low compared to CAF #199C and NAF #200N. Data are mean ± SD of triplicate samples. * P<0.05. *** P<0.001. (TIF) [file pone.0015313.s003.tif]

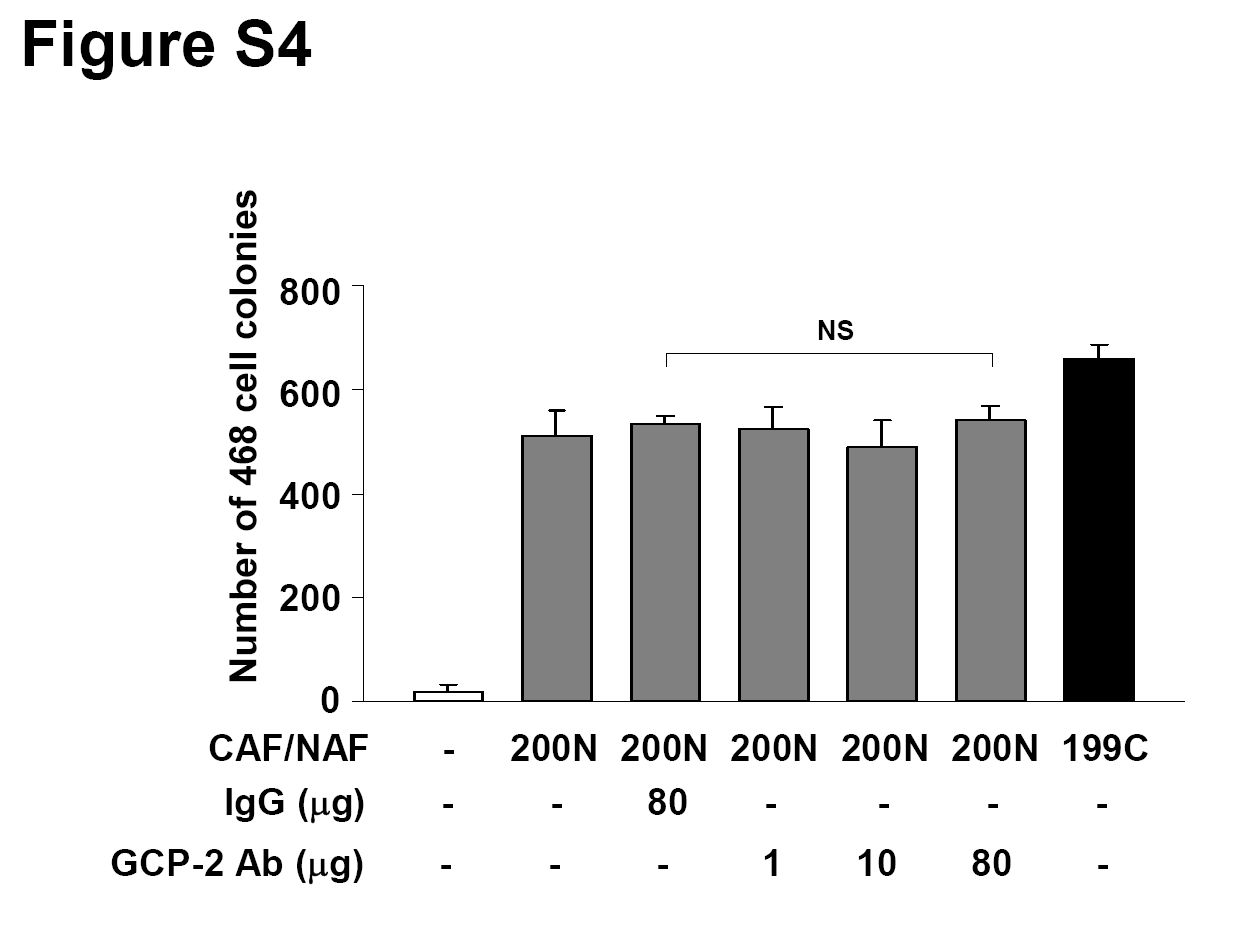

Supplement: Figure S4 — Sequestration of the GCP-2 activity did not affect NAF-mediated soft agar colony formation of the breast cancer MDA-MB-468 cells. NAF #200N-mediated soft agar colony formation of MDA-MB-468 cells was not affected by addition of 80 µg/ml anti-GCP-2 antibody. Data are mean ± SD of triplicate samples. * P<0.05. NS, no significant difference. (TIF) [file pone.0015313.s004.tif]
